# The quantum oscillations in different probe configurations in the $\hbox {BiSbTe}_{{3}}$ topological insulator macroflake

**DOI:** 10.1038/s41598-022-09073-4

**Published:** 2022-03-25

**Authors:** Shiu-Ming Huang, Chien Lin, Sheng-Yu You, You-Jhih Yan, Shih-Hsun Yu, Mitch Chou

**Affiliations:** 1 Department of Physics, National Sun Yat-Sen University, Kaohsiung, 80424 Taiwan; 2 Center of Crystal Research, National Sun Yat-Sen University, Kaohsiung, 80424 Taiwan; 3 Department of Materials and Optoelectronic Science, National Sun Yat-Sen University, Kaohsiung, 80424 Taiwan; 4 Taiwan Consortium of Emergent Crystalline Materials, TCECM, National Sun Yat-Sen University, Kaohsiung, 80424 Taiwan

**Keywords:** Topological insulators, Quantum mechanics

## Abstract

We demonstrate quantum oscillations in $\hbox {BiSbTe}_{{3}}$ topological insulator macroflakes in different probe configurations. The oscillation period in the local configuration is twice compared to the non-local configuration. The Aharonov–Bohm-like (AB-like) oscillation dominates the transport property in the local configuration and the Altshuler–Aronov–Spivak-like (AAS-like) oscillation dominates the transport property in the non-local configuration. The AB-like oscillation period is 0.21 T and the related loop diameter is 156 nm which is consistent with the reported phase coherence length in topological insulators. The Shubnikov–de Haas oscillation frequency is the same but oscillation peaks reveal a $\pi$ phase shift in the local and non-local configuration. The Berry phase is $\pi$ in the local configuration and 0 in non-local configuration.

## Introduction

Quantum interference such as universal conductance fluctuations^1^, Aharonov–Bohm (AB) oscillation^2,3^ and Altshuler–Aronov–Spivak (AAS) oscillation, is a wave characteristic of carrier transport. These oscillations originate from a magnetic field flux, $\Phi _{0}$, through a close loop by two carrier transport trajectories with the same phase in clockwise and counterclockwise directions. The oscillation periodicity of AB and AAS oscillations are related to the of flux quanta, $\frac{h}{e}$ and $\frac{h}{2e}$, where the *h* and *e* are the Planck’s constant and electron charge, respectively. These oscillations are extremely sensitive to the carrier coherence length and are excellent tools to probe carrier transport properties. With this, it is broadly used to investigate intrinsic characteristics in various kinds of nanowires and patterned nano-structures^4–12^. The recent study demonstrates the AB-like oscillation in a topological insulator macroflake^13.^ The intrinsic mechanism between AB and AAS oscillations are different and the magnetic flux quantum are $\frac{h}{e}$ and $\frac{e}{2h}$ for AB and AAS interference, respectively which result to half oscillation frequency difference between AB and AAS interference. These two interferences coexist in carrier transports. It is curious that whether one could individually demonstrate the AAS-like oscillation in a mesoscopic system. It is known that as well as the material intrinsic properties, different carrier transport processes leads to different behaviors. One might construct a particular probe configuration to comprehensively detect and optimize the specific carrier transport characteristics.

In this work, we individually realize the experimental demonstration of AB-like and AAS-like oscillations in different probe configurations in the $\hbox {BiSbTe}_{{3}}$ topological insulator macroflake. The carrier transport properties were extracted from the AB-like and AAS-like oscillations and consistent with theoretical predictions. Furthermore, our experimental result revealed that the Shubnikov-de Haas oscillation frequency was the same but oscillation peaks revealed a $\pi$ phase shift in the local and non-local configurations. The Berry phase is $\pi$ in the local configuration and 0 in non-local configuration.

## Experimental method

Single crystals of $\hbox {BiSbTe}_{{3}}$ were grown using a homemade resistance-heated floating zone furnace (RHFZ). The single crystal grow condition is the same as the previous reports. Our previous work demonstrated that topological insulator (TI) with extremely high uniformity can be obtained using the RHFZ method^13–19^. Energy-dispersive X-ray spectroscopy (EDS) confirmed the stoichiometric ratio of the crystal to be Bi:Sb:Te $= 1:1:3$, while the XRD spectrum confirmed the crystal structure consistent with $\hbox {BiSbTe}_{{3}}$ database.

The cleaved $\hbox {BiSbTe}_{{3}}$ single crystal flakes were obtained using the scotch-tape method. The cleaved flake geometry is roughly 3 mm in length, 2 mm in wide, and 170 $\mu$m in thickness. Gold wires were electrically attached to the cleaved crystal surface using silver paste. The Raman and EDS spectrum support that the crystal is $\hbox {BiSbTe}_{{3}}$. Magnetotransport measurements were performed using the standard 4-probe technique in a commercial apparatus (Quantum Design PPMS) with a magnetic field up to 14 T. The *B* was applied perpendicular to the large cleaved surface. The data points are taken per 100 Gauss at magnetic field region between 6 to 14 T in the steady magnetic field mode, instead of the sweeping magnetic field mode. The data points are taken after the magnetic field is steady at the setting magnetic field for 1 minute.

**Figure 1:**
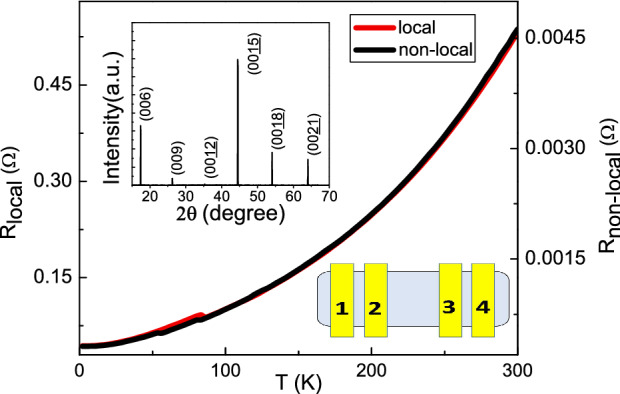
The top-left inset shows the XRD spectrum revealing sharp peaks testifying high quality $\hbox {BiSbTe}_{{3}}$ crystal. The bottom-right inset shows the probe configuration in this work. The applied current, $I_{14}$, flows through the electrode 1 and 4, and the voltage difference, $V_{23}$, is detected at the electrode 2 and 3 in the local configuration. The applied current, $I_{21}$, flows from the electrode 2 to electrode 1, and the voltage difference, $V_{34}$, is detected at the electrode 3 and 4 in the non-local configuration. The resistance, *R*, is determined by the ratio of the detected voltage difference to the applied current in both two configurations. The measured resistances in two configurations follow the same temperature dependence from 300 to 2 K. The residual resistance ratio, $R(2 \mathrm {K})/R(300\, \mathrm {K})$, reaches 0.07.

In this work, we probe the transport characteristics of the $\hbox {BiSbTe}_{{3}}$ in two different probe configurations, the local and the non-local probe configurations. Both probe configurations are four-probe method. As shown in the bottom-right inset of the Fig. 1, the applied current, $I_{14}$, flows through the electrode 1 and 4, and the voltage difference, $V_{23}$, is detected at the electrode 2 and 3 in the local configuration. The applied current, $I_{21}$, flows from electrode 2 to electrode 1, and the voltage difference, $V_{34}$, is detected at the electrode 3 and 4 in the non-local configuration. The resistance, *R*, is determined by the ratio of the detected voltage difference to the applied current in both two configurations. To avoid the signal interference due to frequently electrode switching in two different probe configurations. The non-local configuration is performed after one takes all data in different magnetic fields and temperatures in the configuration. The non-local probe configuration is widely used to detect the carrier characteristics in the diffusion process in various kinds of materials and systems.

## Results and discussion

The top-left inset of Fig. 1 shows the XRD spectrum and it reveals extremely sharp peaks. That supports the $\hbox {BiSbTe}_{{3}}$ is highly crystallized. Figure 1 shows the temperature dependent resistances in the local and non-local measurement configurations both of which showing metallic behavior. Due to the difference of transport mechanisms, the measured resistances in the local configuration is 2-orders higher than that in the non-local configuration. The measured resistances in two configurations follow the same temperature dependence from 300 to 2 K. The residual resistance ratio, $R(2\, \mathrm {K})/R(300\, \mathrm {K})$, reaches 0.07 in two configurations and lower than most reported values in topological insulators. These support the highly quality and uniformity of our $\hbox {BiSbTe}_{{3}}$.

**Figure 2:**
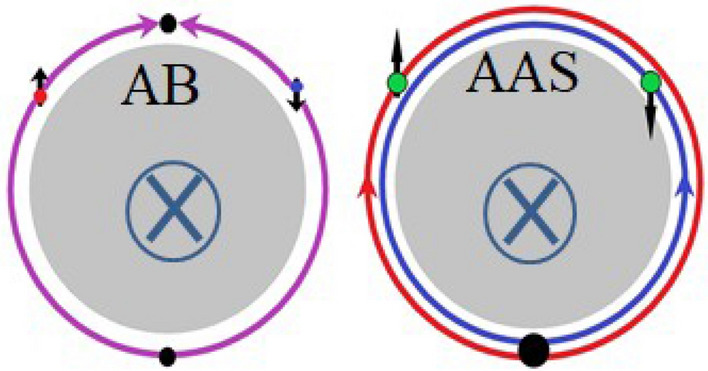
The cartoon of AB and AAS oscillations. Carriers travel half loops and form quantum interference at the other side of the loop in the AB oscillation. Carriers travel a whole loop and form quantum interference at the original position in the AAS oscillation.

The magnetic field, *B*, would lead to phase shift of the carrier wavefunction in the loop which is patterned by two carriers with different count-directions and that leads to AB and AAS interferences as shown in Fig. 2. The AB interference leads to periodic conductance oscillation on the basis of a magnetic flux quantum, $\Phi _{0}=h/e$. The *h* and *e* are the Plank’s constant and electron charge, respectively. The AB oscillation period is expressed as $\Delta B = \Phi _{0}/A$ where *A* is the loop area^10^. The AAS interference originates from a pair of time reversal loops and the oscillation is on the basis of a magnetic flux quantum, $\Phi _{0}=h/2e$. It is similar to the AB interference but the oscillation period is half of AB oscillation. It is worthy to pay attention on that carriers travel half loops and form quantum interference at the other side of the loop in the AB oscillation, and carriers travel a whole loop and form quantum interference at the original position in the AAS oscillation.

**Figure 3:**
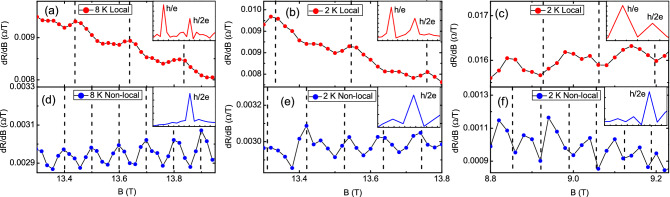
The magnetoresistance as a function of magnetic fields. (**a**–**c**) The *dR*/*dB* in local configuration, and (**d**–**f**) the *dR*/*dB* in nonlocal configuration. The oscillation period in the local configuration is double of that in the nonlocal configuration.

Magnetoresistances are performed in local and non-local configurations. Figure 3 shows the derivative resistance with respected to the applied magnetic field, *dR*/*dB*, as a function of magnetic field in both local and non-local configurations with temperature and magnetic field as the tuning parameter. It shows periodic oscillations in both configurations. The oscillation period in local configuration is double of that in the non-local configuration. The *dR*/*dB* oscillation amplitude in the local configuration is larger than that in the non-local configuration.

To confirm and identify the intrinsic mechanism of the observed different oscillation periods in two configurations, the fast Fourier transform was performed. As shown in the inset of Fig. 3, there are two oscillation peaks in the local configuration and only one peak in the non-local configuration. The second oscillation frequency is double of the first oscillation frequency in the local configuration. The oscillation period is about 0.21 T in the local configuration and the related loop area is 1.92 $\times 10^{-14}$ $\hbox {m}^{2}$. The corresponding diameter is 156 nm which is consistent with the reported phase coherence length for topological insulators. That supports that the observed first oscillation peak of 5 $\hbox {T}^{-1}$ in the local configuration might originate from the AB interference. Different from the conventional AB oscillation in patterned nano structures or nanowires, the observed AB-like oscillation is speculated to be originated from a series connected elastic scattering trajectory loop in a macroflake. These oscillations are corresponding to the AB-like (*h*/*e*) and AAS-like (*h*/2*e*) interference. The oscillation peak in the non-local configuration is the same as the peak position of second oscillation peak in the local configuration. The AB-like oscillation is diminished and only AAS-like oscillation is observed in the non-local configuration. The weak AAS signal was often covered by AB signal and one rarely detects the sole AAS signal in the conventional probe configuration. Our result revealed that one can individually detect the AB-like and AAS-like interference using different probe configurations. The theoretical calculation supports that one could rule out the smearing effect from the interference from loops with different sizes due to the different contribution loop numbers with different size^20^. The observed single peak originates from the largest loop size which is related to the carrier coherence length.

**Figure 4:**
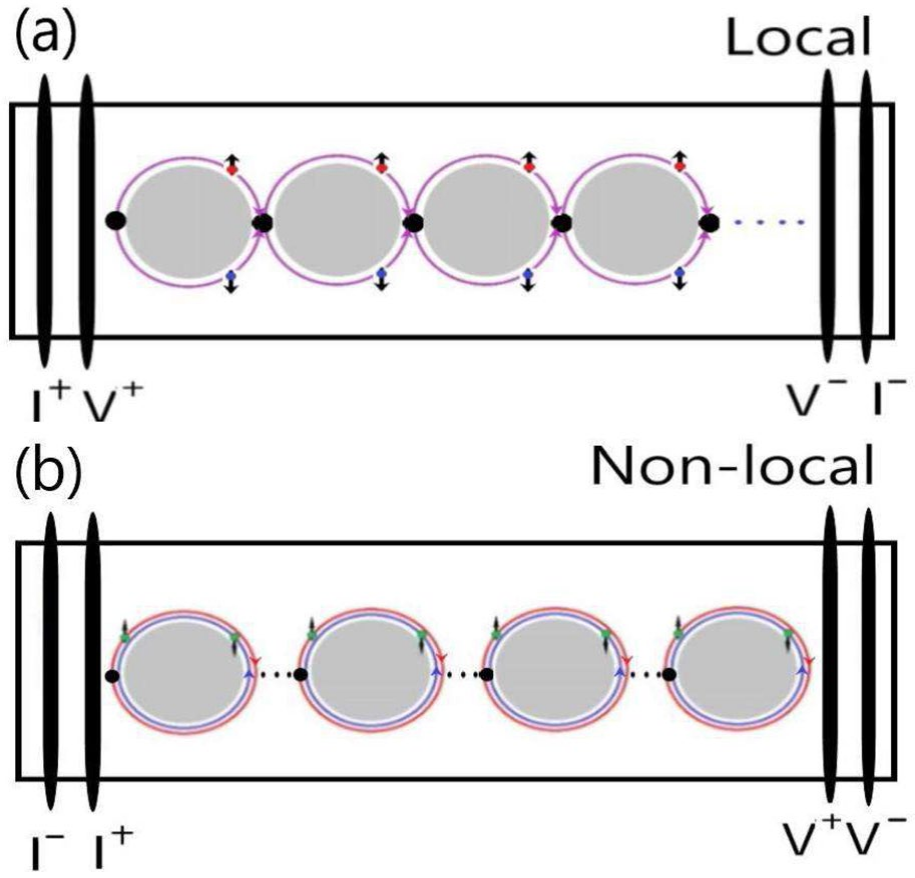
The cartoons of AB-like and AAS-like interferences. (**a**) The AB-like interference is dominant in the local configuration. (**b**) the AAS-like interference is dominant in the nonlocal configuration.

It is theoretically argued that the AAS-like interference would be suppressed in the case of spin-helical carriers with opposite spins in topological insulators^8,10^. Our experimental observation revealed clear AAS-like oscillations from surface state carriers. This supports that carriers with spin-helical texture would not eliminate the AAS-like interference. One question arises as to why the AB-like interference gets suppressed while the AAS-like survives in the non-local configuration? Figure 4 shows cartoons of AB-like and AAS-like interferences in a macroflake system with different configurations. Carriers travel half loops and form quantum interference at the other side of loops in the AB-like oscillation. Carriers travel a whole loop and form quantum interference at the original position in the AAS-like oscillation. The sample in this work is in the order of mm which is much longer than the carrier phase coherence length. As shown in Fig. 4, carrier trajectory forms a series of connected AB-like interference loops. The effective quantum oscillation signal is directly related to the combination of these loops. Without the external voltage, carriers would form random-connected AB-like loops in the non-local configuration. The effective loop number would greatly increase in the diffusion process. Following the Landauer–Büttiker formula in which the detected AB-like signal would be greatly suppressed^21^. On the other hand, AAS-like interference originates from a pair of time-reversal loops. The carrier phase shift by the scattering during the two reverse loops is the same, therefore, the AAS-like would survive the environment scattering^22^. The AAS-like is predicted to be dominant in systems with strong disorders and solely depends on the phase coherence length^23^. These AAS-like loops could individual exist in a mesoscopic system, thus the AAS-like signal is tolerant to the loop number effect.

The previous experiment revealed that the AB oscillation frequency were consistent in the local and non-local configurations in an asymmetric quantum ring^5^. The ring geometry is 2 $\mu$m that is close to the carrier elastic scattering length. Similar observation is reported in patterned nano-circuits^6^. As we mentioned in the discussion, the signal reduction originates from the massive random-connected quantum loops. These reported focus on their works on the patterned geometry which confines the carrier transport trajectory and that might weaken the loop number effect in the diffusion process. It is worthy to emphasize that no obvious AAS interference signal is observed in both configurations in the asymmetry rings. That might originate from the asymmetry patterned structure and/or weak AAS oscillation signal in the system.

**Figure 5:**
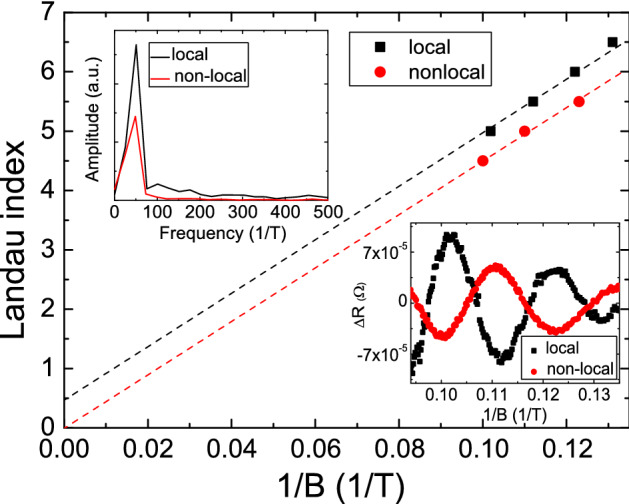
The bottom-right inset shows the extracted SdH oscillations in both configurations. The oscillation shifts a $\pi$ phase in the two configurations. The top-left inset shows the FFT of SdH oscillation in two configurations. The oscillation peak positions are the same. The Landau level fan diagram of two SdH oscillation. The Berry phase are $\pi$ in the local configuration and 0 in the nonlocal configuration.

The bottom-right inset of Fig. 5 shows the extracted magnetoresistances as a function of 1/*B* in local and non-local configurations. It reveals periodic oscillations in two configurations and is known as Shubnikov–de Haas (SdH) oscillations. It is interesting that oscillation peaks revealed a $$\pi$$ phase shift in the local and non-local configurations. The SdH oscillation arises from the successive emptying of Landau levels with the increase of *B*, expressed as $$\frac{1}{B} = \frac{2\pi e}{\hbar A_{F}} (N+\beta ),$$ where $$A_{F}=\pi k_{F}^{2}$$ is the cross section area of the Fermi surface, $$k_{F}$$ is the Fermi wave vector, *N* is the Landau level and $$\beta$$ is the Berry phase^24,25^. The top-left inset shows the fast Fourier transform of SdH oscillations and a sharp peak at 52 $$\hbox {T}^{-1}$$ in both configurations^26^. Following the Onsager relation $$F=\frac{\hbar A_{F}}{2\pi e}$$, where *F* is the SdH oscillation frequency, the corresponding Fermi wavevector, $$k_{F}$$, is 3.9 $$\hbox {nm}^{-1}$$ that is consistent with reported $$k_{F}$$ of surface state in $$\hbox {BiSbTe}_{{3}}$$ topological insulators^26^. That supports that these SdH oscillations originate from surface state carriers in our $$\hbox {BiSbTe}_{{3}}$$ topological insulator. The $$\beta$$ could be interfered from the Landau level fan diagram. Figure 5 shows the Landau level fan diagram with magnetoresistance peaks and dips assigned to Landau level *N* and $$N+0.5$$, respectively. The intercept is 0.5 that indicates the $$\beta$$ is $$\pi$$ in the local configuration and the intercept is 0 that indicates $$\beta$$ is 0 in the non-local configurations. The topological insulator surface state carrier is a Dirac Fermion with a Berry phase of $$\pi$$. Our observation revealed that the different probe configuration would diminish the transport characteristics of Berry phase. Similar behavior is observed in the Dirac semimetal $$\hbox {Cd}_{{3}}$$$$\hbox {As}_{{2}}$$ nanoplates^27^, topological insulator nanoribbon^28^, and AlGaAs/GaAs heterostructure^5,29^. That might originate from the transport characteristic of diffusion process in the non-local configuration. It needs further investigation to clarify the detail mechanic of the $$\pi$$ phase shift in different probe configurations.

## Conclusion

We demonstrate quantum oscillations in $\hbox {BiSbTe}_{{3}}$ topological insulator macroflakes in different probe configurations. The oscillation period in the local configuration is double of that in the non-local configuration. The Aharonov–Bohm-like (AB-like) oscillation dominates the transport property in the local configuration and the Altshuler–Aronov–Spivak-like (AAS-like) oscillation dominates the transport property in the non-local configuration. The AB-like oscillation period is 0.21 T and the related loop diameter is 156 nm which is consistent with the reported phase coherence length in topological insulators. The Shubnikov–de Haas oscillation frequency is the same but oscillation peaks revealed a $\pi$ phase shift in the local and non-local configuration. The Berry phase is $\pi$ in the local configuration and 0 in non-local configuration.
